# Therapeutic Benefit of Systematic Lymphadenectomy in Node-Negative Uterine-Confined Endometrioid Endometrial Carcinoma: Omission of Adjuvant Therapy

**DOI:** 10.3390/cancers14184516

**Published:** 2022-09-17

**Authors:** Isao Otsuka

**Affiliations:** Department of Obstetrics and Gynecology, Kameda Medical Center, Kamogawa 296-8602, Chiba, Japan; otsuka.isao@kameda.jp

**Keywords:** endometrioid endometrial carcinoma, systematic lymphadenectomy, pelvic and para-aortic lymphadenectomy, intermediate-risk, high-risk, node-negative, adjuvant therapy

## Abstract

**Simple Summary:**

Endometrial cancer is the most common gynecological tract malignancy in developed countries. Extrauterine disease, in particular lymph node metastasis, is an important prognostic factor. Nevertheless, pelvic lymphadenectomy is not considered to have a therapeutic benefit, as it did not improve survival in randomized studies. However, lymphadenectomy may have a therapeutic benefit if adjuvant therapy can be omitted without decreasing oncological outcomes, as the long-term quality of life is maintained by avoiding morbidities associated with adjuvant therapy. In intermediate- and high-risk endometrioid endometrial carcinomas, adjuvant therapy may be safely omitted without decreasing long-term survival by open surgery including systematic pelvic and para-aortic lymphadenectomy when patients are node-negative. Systematic lymphadenectomy may remove undetectable low-volume lymph node metastasis in both pelvic and para-aortic regions, and open surgery may reduce vaginal recurrence even without vaginal brachytherapy. However, lymphadenectomy may not improve survival in elderly patients and patients with p53-mutant tumors.

**Abstract:**

Endometrial cancer is the most common gynecological tract malignancy in developed countries, and its incidence has been increasing globally with rising obesity rates and longer life expectancy. In endometrial cancer, extrauterine disease, in particular lymph node metastasis, is an important prognostic factor. Nevertheless, pelvic lymphadenectomy is not considered to have a therapeutic benefit, as it did not improve survival in randomized studies. However, lymphadenectomy may have a therapeutic benefit if adjuvant therapy can be omitted without decreasing oncological outcomes, as the long-term quality of life is maintained by avoiding morbidities associated with adjuvant therapy. In intermediate- and high-risk endometrioid endometrial carcinomas, adjuvant therapy may be safely omitted without decreasing long-term survival by open surgery including systematic pelvic and para-aortic lymphadenectomy when patients are node-negative. Systematic lymphadenectomy may remove undetectable low-volume lymph node metastasis in both pelvic and para-aortic regions, and open surgery may reduce vaginal recurrence even without vaginal brachytherapy. However, lymphadenectomy may not improve survival in elderly patients and patients with p53-mutant tumors. In this review, I discuss the characteristics of lymph node metastasis, the methods of lymph node assessment, and the therapeutic benefits of systematic lymphadenectomy in patients with intermediate- and high-risk endometrioid endometrial carcinoma.

## 1. Introduction

Endometrial cancer is the most common female genital tract malignancy in developed countries. Its incidence has been increasing globally because of rising obesity rates and longer life expectancy [1,2]. The majority of endometrial cancers are confined to the uterus, and patients with such diseases have a favorable prognosis. In contrast, patients with extrauterine disease, particularly lymph node metastasis, have a poorer survival. To assess lymph node status, lymphadenectomy or sentinel lymph node biopsy is performed. Lymphadenectomy has a diagnostic benefit by providing the knowledge of pathological lymph node status that is useful for tailoring the appropriate adjuvant therapy. However, pelvic lymphadenectomy has not been thought to have a therapeutic benefit, as it did not improve survival in two randomized controlled trials [3,4]. Thus, attempts have been made to reduce surgical morbidity of lymphadenectomy with the introduction of sentinel lymph node biopsy.

In contrast, attempts to reduce the use of adjuvant therapy in the management of endometrial cancer have been limited, although its effect on overall survival, similar to that of lymphadenectomy, has not been established [5,6,7,8,9]. In node-positive patient adjuvant therapy, radiation therapy and/or chemotherapy is given to eradicate residual diseases after surgery. However, even though lymph node metastasis is not detected by lymph node assessment, adjuvant therapy is recommended for patients with uterine-confined disease considered to be at risk of recurrence under current treatment guidelines [10,11,12].

Lymphadenectomy may have a therapeutic benefit in node-negative patients if adjuvant therapy can be omitted without decreasing oncological outcomes, because morbidities that decrease long-term quality of life of survivors associated with adjuvant therapy can be avoided [13,14,15,16,17,18,19]. In this review, I discuss the characteristics of lymph node metastasis in endometrial carcinoma, the methods of lymph node assessment, and the possibility of omitting adjuvant therapy in patients with node-negative uterine-confined endometrioid carcinoma who undergo systematic lymphadenectomy. In this review, I focus on endometrioid endometrial carcinoma, in particular intermediate- and high-risk diseases. A detailed discussion of the high-risk histological subtypes, such as serous carcinoma and carcinosarcoma, is beyond the scope of this review.

## 2. What Is the True Incidence of Lymph Node Metastasis?

Endometrial cancers can be classified into low-, intermediate-, and high-risk diseases based on the risk of recurrence (Table 1) [5,6,10,11,12]. The risk of recurrence varies according to uterine pathological factors, such as depth of myometrial invasion, tumor grade, cervical invasion, and lymphovascular space invasion [20]. Although the low-risk disease is almost identical, i.e., stage IA, Grade 1 or 2 endometrioid carcinoma, the definitions of intermediate- and high-risk disease are different by group. High-intermediate risk disease may be the same as high-risk disease without gross extracorporeal disease. The risk of recurrence, other than vaginal recurrence, is almost identical to that of lymph node metastasis [21].

Although the uterus is a pelvic organ, endometrial cancer metastasizes to both the pelvic and para-aortic lymph nodes. Pelvic lymph node metastasis is more often observed; however, para-aortic node metastasis is not rare [22,23,24,25,26]. In our previous study, lymph node metastasis was observed in 27 (9.2%) of the 292 patients with endometrioid endometrial carcinoma; pelvic and para-aortic node metastases were observed in 22 (7.5%) and 8 (2.7%) patients, respectively [26]. Most para-aortic node metastases develop in patients with pelvic node metastasis [22,24,26,27,28,29]. However, isolated para-aortic node metastasis is also observed [3,20,26,29,30,31,32,33,34].

Metastatic carcinomas in the lymph nodes are classified according to their size as macrometasasis (larger than 2 mm in diameter), micrometastasis (0.2 mm to 2 mm), and isolated tumor cells (smaller than 0.2 mm) [35,36]. As low-volume disease, i.e., micrometastasis and isolated tumor cells, may not be detected by standard pathological assessment, ultrastaging using serial sections of the nodes and immunohistochemical staining are necessary.

Thus, the true incidence of lymph node metastasis in endometrial carcinoma cannot be determined unless all pelvic and para-aortic lymph nodes are removed by systematic lymphadenectomy and pathologically assessed using the ultrastaging method. The incidences of lymph node metastasis detected by standard pathological evaluation have been underestimated.

## 3. Role of Adjuvant Therapy in Endometrial Cancer

There is no definitive evidence that adjuvant therapy improves the long-term survival of women with endometrial cancer apparently confined to the uterus. Randomized controlled trials have shown that external beam radiotherapy to the pelvis decreases local recurrence but does not improve overall survival [5,6,7]. Although external beam radiotherapy might improve survival in patients with high-intermediate risk disease [6], a meta-analysis indicated that in intermediate- and high-risk patients, adjuvant external beam radiotherapy did not improve overall survival [37]. In intermediate-risk disease, adjuvant vaginal brachytherapy is recommended to avoid gastrointestinal complications that are often associated with external beam radiotherapy [10,11,12,38]. In node-negative patients with deeply invasive grade 3 endometrioid tumors, no survival benefit was observed with pelvic radiation compared to vaginal brachytherapy alone [39]. However, the necessity of adjuvant vaginal brachytherapy is questioned, because most vaginal recurrences can be cured with salvage therapy [40,41,42].

Adjuvant chemotherapy in patients with high-risk stage I–II endometrioid carcinoma improved survival in one study [43], but did not in another study [44]. In high-intermediate and high-risk early-stage endometrial carcinoma, pelvic and para-aortic nodal recurrences were more common after chemotherapy and vaginal brachytherapy compared to pelvic radiation therapy [45]. Effects of chemotherapy may be limited in grade 3 tumors [46,47].

Late complications caused by adjuvant therapy may reduce the long-term quality of life of survivors. After external beam radiotherapy, gastrointestinal complications may lead to limitations in daily activities [38]. In particular, pelvic radiotherapy following surgery including lymphadenectomy significantly increased risk of serious complications compared to surgery alone [6]. Vaginal brachytherapy may cause vaginal atrophy and subsequent stenosis [17] and severe bowel complications such as rectal bleeding [16]. Chemotherapy including platinum and/or taxane often causes peripheral neuropathy that decreases quality of life [18,19].

Importantly, adjuvant therapy might not replace surgical removal as a treatment for positive nodes in some patients, as patients who underwent lymphadenectomy had an improved survival compared to those who did not undergo the procedure, even though adjuvant therapy was performed [48,49,50,51].

Theoretically, adjuvant therapy is not necessary for patients with node-negative uterine-confined disease, because residual diseases that otherwise need to be eradicated by adjuvant therapy are not left behind. Patients with clinically negative but histologically positive nodes can also become disease-free by both lymphadenectomy and/or sentinel lymph node biopsy followed by lymphadenectomy.

## 4. Sentinel Node-Negative Patients: Can Adjuvant Therapy Be Omitted?

Sentinel lymph node mapping has been increasingly performed instead of lymphadenectomy that may be associated with surgical morbidities and sequelae, such as vascular injury and lymphedema [52,53]. The sentinel lymph node is defined as the first node in the lymphatic basin that receives drainage from the primary tumor. If the sentinel lymph node is negative, a regional lymphadenectomy can be avoided [54]. In breast cancer and melanoma, both of which are superficial cancers with less complicated lymphatic drainage routes, the sentinel lymph node technique has been incorporated into standard practice [55,56]. In endometrial cancer, sentinel lymph node mapping has a high degree of diagnostic accuracy and detects low-volume metastasis in the sentinel nodes [57,58,59,60,61].

However, in intermediate- and high-risk endometrial cancers, the detection of the true sentinel nodes by the standard method, cervical injection of a tracer, may be difficult. Whereas low-risk diseases metastasize almost only to pelvic nodes, intermediate- and high-risk diseases metastasize to not only pelvic nodes, but also para-aortic nodes. The lymphatic network draining the uterus is complex and involves both pelvic and para-aortic nodes [62], as lymphatic channels draining from the uterine fundus course into the broad ligament and along the ovarian vessels [62,63]. The incidence of isolated para-aortic node metastasis (without pelvic node metastasis) in patients with intermediate- and high-risk diseases ranges from 2.6 to 3.1% [26,29,33], and the majority of para-aortic node metastases are observed above the inferior mesenteric artery [23]. Cervical injection is superior in detecting pelvic sentinel nodes [64] and achieves a higher sentinel node detection rate than fundal injection [65,66]. However, hysteroscopic or ultrasound-guided fundal sub-endometrial injection is necessary to detect para-aortic sentinel nodes [66,67,68,69,70,71].

Histopathological detection of metastatic lesions in the sentinel lymph nodes may be difficult during surgery. As a lymph node metastasis less than 2 mm was often missed at frozen section examination [72], ultrastaging is necessary to detect the majority of low-volume metastases in the sentinel nodes that account for 25–48% of sentinel node metastasis [57,73,74,75,76]. Many studies testing the sensitivity of sentinel lymph nodes have reported high sensitivity; however, in most studies, pathological evaluation of sentinel nodes was performed after back-up lymphadenectomy following sentinel lymph node biopsy [59,61,65,73,77,78,79,80].

Most importantly, negative sentinel nodes may lead to the omission of lymphadenectomy, but may not necessarily lead to the omission of adjuvant therapy. A false negative rate of 15% has been reported with a blue dye cervical injection [81]. Current studies on sentinel lymph node mapping were aimed at the accurate detection of metastatic lymph nodes by performing back-up lymphadenectomy; thus, lymphadenectomy was rarely omitted [59,61,65,73,77,78,79,80]. Adjuvant therapy was given even in patients with node-negative uterine-confined disease when they were considered to be at risk of recurrence based on uterine pathological factors [82,83]. In node-negative deeply invasive endometrioid carcinoma, patients who underwent sentinel lymph node mapping were more likely to receive adjuvant therapy than patients who underwent lymphadenectomy [84]. A previous study reported that of 54 sentinel node-negative patients treated with adjuvant therapy, eight (15%) developed recurrence [77]. If adjuvant therapy is given according to uterine pathological factors irrespective of nodal status, sentinel lymph node mapping, which significantly increases the costs compared to hysterectomy alone [53], might not be necessary. To evaluate the efficacy of sentinel lymph node biopsy, prospective studies evaluating the long-term oncologic outcome are necessary [85], particularly the long-term safety of omitting both systematic lymphadenectomy and adjuvant therapy in sentinel node-negative patients. Long-term follow-up is indispensable to detect lymph node recurrence, because it often develops five years or later [86].

## 5. Benefit of Systematic Lymphadenectomy in Node-Negative Patients: Omission of Adjuvant Therapy

For an accurate evaluation of the therapeutic effects of lymphadenectomy, appropriate patients need to undergo appropriate surgery, as selection of appropriate patients is necessary to detect survival difference between patients who undergo lymphadenectomy and those who do not. First, patients at low-risk for lymph node metastasis need to be excluded [87,88,89]. In these patients, the incidence of lymph node metastasis is at most 5.9%, when considering low-volume metastasis [74]; thus, survival difference cannot be detected without a sufficient power. It is plausible that effects of lymphadenectomy on survival cannot be detected in randomized trials, considering the rate of low-risk disease in endometrioid endometrial carcinoma [3,4,90,91]. Second, patients with high-risk histological subtypes, i.e., serous carcinoma and carcinosarcoma, also need to be excluded, as they often develop peritoneal and hematogenous spread independent of lymph node metastasis [92,93,94].

In intermediate- or high-risk endometrioid carcinomas, systematic lymphadenectomy, particularly pelvic with para-aortic lymphadenectomy, appears to improve survival [50,95,96,97,98,99,100]. Incidences of lymph node metastasis in intermediate- and high-risk endometrioid carcinomas were 17% and 25%, respectively, in our previous study [26]. These incidences may be underestimated, and when ultrastaging was performed, the incidence of lymph node metastasis in high-intermediate risk endometrioid endometrial carcinoma was 24–43% [101,102].

Additionally, for lymphadenectomy to have a therapeutic benefit, no positive lymph nodes are left behind at the completion of surgery. Namely, it is necessary to remove lymph nodes bearing not only macroscopic but also low-volume metastases. Moreover, lymph nodes in the para-aortic region as well as those in the pelvic region need to be removed in intermediate- and high-risk endometrial carcinoma when deep myometrial invasion is observed [103].

However, the procedures performed during lymphadenectomy range from a mere sampling of enlarged or suspicious lymph nodes for staging purposes to systematic removal of all accessible lymphatic tissue with a therapeutic intent [88]. Systematic complete lymphadenectomy is performed to remove enlarged nodes and to skeletonize the vessels of node-bearing tissue [30,88]. The number of nodes removed varies among studies: the median numbers of nodes removed in the pelvic and para-aortic region were in the ranges of 11–54 and 5–23, respectively [26,29,95,97,98,103,104]. The quality of surgical resection may be measured by a nodal count that is indicative of the extent of nodal dissection, although the number of nodes reported by the pathologist depends on surgical expertise, the comprehensiveness of pathological analysis, and anatomical variations in patients [88]. The removal of 10 or more regional lymph nodes was associated with improved survival in intermediate- and high-risk endometrioid carcinomas [48,49,105]. The number of nodal stations sampled may be a more accurate predictor of lymph node metastasis than lymph node count [106,107].

The number of nodes removed is associated with improved survival also in patients with other cancers, i.e., breast, lung, and cervical cancers, even when all regional lymph nodes are interpreted as pathologically negative [108,109,110,111]. In particular, with the removal of larger numbers of nodes, regional relapse was significantly decreased for breast cancer patients not receiving systemic therapy [109].

Node-negative patients consist of true node-negative and false node-negative patients. False node-negative patients have occult lymph node metastasis that cannot be detected by standard pathological evaluation or that cannot be removed by non-systematic lymphadenectomy.

Systematic lymphadenectomy can remove all lymph nodes bearing macroscopic and low-volume metastases, as patients with endometrioid carcinoma who undergo systematic lymphadenectomy rarely develop lymph node recurrence, even without adjuvant therapy [26,30,112,113,114]. Similarly, nodal recurrences were rare in patients who underwent systematic lymphadenectomy with subsequent vaginal brachytherapy alone [104,115,116,117].

More importantly, in intermediate- and high-risk endometrioid endometrial carcinomas, adjuvant therapy may be omitted without decreasing survival by open surgery with systematic pelvic and para-aortic lymphadenectomy when patients are node-negative (Table 2) [26,112,113,114]. In our prospective cohort study of 77 node-negative patients with intermediate- and high-risk endometrioid carcinoma, only two understaged high-risk patients died of disease [26]. Although hematogenous spread (pulmonary metastasis is most commonly observed) may develop in endometrioid carcinoma, its risk is low in patients without extrauterine diseases [118,119]. Thus node-negative patients undergoing systematic lymphadenectomy that can remove all lymph nodes including positive nodes containing undetectable low-volume metastasis may be considered to be true node-negative. In contrast, sentinel node-negative patients without back-up lymphadenectomy might have a higher possibility of having undetected residual lymph node metastasis at the completion of surgery. Of note, in patients with positive peritoneal cytology, adjuvant therapy may not be omitted, as positive peritoneal cytology in low-stage disease was associated with decreased survival [120].

Node-positive patients, in particular patients with low-volume metastasis, can be cured with surgery alone including systematic lymphadenectomy. A previous study has reported that five-year overall survival was 40% for node-positive patients treated with surgery alone [121]. We have experienced the long-term survival of a case with grade 3 endometrioid endometrial carcinoma with para-aortic node metastasis treated with surgery alone [122]. She had a pelvic node, a para-aortic node, and an adnexal metastasis, but all metastatic diseases were micrometastasis (<2 mm). Similarly, isolated tumor cells detected in removed sentinel nodes may not decrease survival, even without adjuvant therapy [123,124,125,126].

The patient age, which has been incorporated into the existing risk classification systems [5,6], may influence the effect of lymphadenectomy in endometrioid endometrial carcinoma (Table 3). In elderly women, lymphadenectomy may be less effective than in younger women, which might be indicated in German population-based studies where median age of the patients was 69 years or older [127,128]. This may be explained by the association of older age with adverse pathologic features [129,130], hematogenous dissemination [118], and immunosenescence [131]. Otherwise, the route of surgical approach, which was not described in some recent studies, might affect the results.

## 6. Patients Treated with Minimally Invasive Surgery: Can Adjuvant Therapy Be Omitted?

Minimally invasive surgery, laparoscopic or robot-assisted surgery, has been accepted as a standard surgical approach after two randomized controlled trials [132,133], as minimally invasive surgery has advantages including shorter hospital stays and lower surgical morbidity compared to open surgery. However, no firm evidence that the effect of minimally invasive surgery on long-term survival in intermediate- and high-risk patients is equivalent to that of open surgery has been established [132,133]. A randomized trial where lymphadenectomy was performed in all patients failed to show the non-inferiority of laparoscopy [132]. Another randomized trial showed that the use of open surgery and laparoscopic surgery resulted in equivalent survival outcomes [133]. However, in that study, only selected surgeons performed surgery on a highly selected group of endometrioid carcinoma patients [134]. A previous study showed that patients treated with minimally invasive surgery had a shorter recurrence-free survival than those treated with open surgery, though overall survival was similar between the two groups [135].

Postoperative recurrences, vaginal and intra-abdominal, may increase in patients treated with minimally invasive surgery, although patients treated with minimally invasive surgery are more likely to receive adjuvant pelvic radiotherapy [136]. Even low-risk patients develop vaginal recurrences after laparoscopic hysterectomy [137]. Cervical involvement was a higher risk of recurrence in patients with intermediate-risk diseases [138]. In patients with high-intermediate risk tumors, minimally invasive surgery was associated with a shorter time to and a higher risk of recurrence [139]. In high-grade endometrial cancers, extracting a large uterus is associated with an increased risk of intra-abdominal recurrence [140]. A recent study reported that robotic surgery was significantly associated with a higher recurrence rate in stage I intermediate-risk endometrioid carcinoma compared to laparotomy [141]. Of note, robotic surgery was significantly associated with poorer overall survival compared to laparoscopic surgery in stage I endometrial cancer [142].

Tumor spillage into the pelvic cavity and the vagina during surgery appears to cause an increased risk of recurrence associated with minimally invasive surgery in patients with endometrial cancer, as well as cervical cancer. Most gynecologic oncologists performing minimally invasive surgeries have experienced uterine perforation with an intra-uterine manipulator and tumor spill while making a colpotomy [143]. Minimally invasive surgery was associated with a higher incidence of positive peritoneal cytology [144]. In patients with polypoid, larger-size tumors or tumors involving the endocervix, vaginal smears collected during surgery were positive for tumor cells in 80% of the patients [145]. A healing wound provides a favorable environment for tumor cells to attach and grow [146], and the leakage of insufflation gas through the vagina and the impact of pneumoperitoneum on local immune reactions may also be associated with tumor development [147]. The poorer outcomes in patients treated with robot-assisted surgery compared to patients treated with laparoscopic surgery may be explained by prolonged operating times, Trendelenburg positioning, and the absence of tactile sensation on tissue manipulation [142]. In cervical cancer, minimally invasive surgery was significantly associated with decreased overall survival, both in a randomized trial and an epidemiological study [148,149]. Even among patients with prostate cancer and colorectal cancer where minimally invasive surgery is commonly used and rarely associated with peritoneal recurrence, patients with positive surgical margins and serous invasion developed peritoneal recurrence possibly due to tumor spillage [150,151]. Prevention of cancer cell dissemination and metastatic tumor formation is a major goal of cancer surgery.

Lower risk of recurrence associated with open surgery may partly be explained by the lower incidence of tumor spillage, because an intra-uterine manipulator that might be associated with an increased risk of recurrence [152,153] is not necessary. In addition, vaginal disinfection after colpotomy during open surgery may prevent tumor implantation. We perform vaginal disinfection using povidone-iodine, which has a cytotoxic effect on tumor cells even in low concentrations [154,155].

Although tumor recurrences after minimally invasive surgery may not decrease overall survival, comorbidities associated with salvage treatment may decrease quality of life in survivors.

However, in view of the ongoing obesity epidemic in some countries, in obese women, minimally invasive surgery appears a better surgical approach to reduce surgical complications [156,157]. In a randomized trial, minimally invasive surgery was associated with a better survival than open surgery in obese women, but the trend failed to reach statistical significance (*p* = 0.14). Conversely, non-obese women tended to have a worse long-term disease-free survival when treated with minimally invasive surgery (*p* = 0.06) [133]. Approximately 10% of patients with early-stage endometrial cancer are medically inoperable because of obesity-related comorbidities, such as cardiovascular disease and diabetes-related end-organ damage [158]. Morbidly obese women are more likely to die of their comorbidities and also of their endometrial cancers [159,160,161]. In women with high-risk disease, severe obesity was associated with a poorer recurrence-free survival, which may be a result of incomplete surgical staging and adapted adjuvant therapies [162].

## 7. Preoperative Prediction of Lymph Node Metastasis and Surgical Therapy

Preoperative detection of lymph node metastasis using imaging studies, i.e., computed tomography, magnetic resonance imaging (MRI), and positron emission tomography/computed tomography, is of limited value, because imaging studies have limited efficacy in detecting tumor cells in small lymph nodes. In contrast, a combination of MRI parameters of tumor, serum CA-125 levels, and tumor grade appears useful in identifying patients in whom lymphadenectomy can be safely omitted [163,164].

Preoperative risk classification using tumor grade may not be accurate, as the agreement between preoperative biopsy and final post-hysterectomy diagnosis for tumor grade was only modest in low-grade tumors (G1 or G2 endometrioid carcinoma) [165], whereas it was high in high-grade tumors (G3 endometrioid, serous, clear cell carcinoma, and carcinosarcoma) [166]. Clinically relevant upgrading from low grade to high grade was observed in 8%, specifically 4% of preoperative grade-1 samples and 14% of preoperative grade-2 samples [165]. In contrast, hormone receptor status determined by preoperative biopsy, i.e., loss of progesterone receptor (PR) and double loss of estrogen receptor and PR, were associated with lymph node metastasis [167,168].

Similarly to chemotherapy, surgical therapy should be individualized based on the molecular profiles of the tumor [169,170]. Patients with *POLE* exonuclease domain mutation (EDM) may not need to undergo lymphadenectomy, as none of the *POLE* EDM tumors had extrauterine disease [171]. Lymph node metastasis was observed in 45% of p53-mutant tumors [172], although p53 abnormality may not be accurately detected in preoperative biopsy specimens, as preoperative biopsy did not show abnormal p53 staining in 56% of the tumors that showed p53 overexpression in hysterectomy specimens [167]. Lymphadenectomy may not improve patients with p53-mutant tumors, as the poor prognosis of early-stage p53-mutant endometrial cancers [173,174,175,176] may not be due to undetected lymph node metastasis [175]. This may be associated with a high proportion of serous carcinoma in p53-mutant tumors, with 71–88% of serous carcinomas being p53 abnormal [175,177], and serous carcinomas often develop peritoneal dissemination [92]. In endometrioid carcinomas, p53 abnormality was also observed in 3%, 11%, and 26–37% of grade-1, grade-2, and grade-3 tumors, respectively [175,177]. A small group of endometrial cancers harbor more than one molecular classifying feature (multiple classifier), and in grade-3 endometrioid carcinoma with mismatch repair deficiency or *POLE* EDM, hence coexisting *TP53* mutation may be a secondary event acquired during tumor progression and was not associated with poorer survival [178]. Thus, p53 abnormality detected with immunohistochemistry may not be associated with poorer survival in patients with node-negative uterine-confined endometrioid carcinoma who undergo systematic lymphadenectomy.

High-intermediate-risk tumors are associated with lymph node metastasis [100,179]. In patients with high-intermediate-risk disease, 15% had unfavorable features such as p53-mutant, 50% favorable features such as *POLE*-mutant, and 35% intermediate features such as microsatellite instability [180]. Molecular profiling revealed that women with *POLE* mutant tumors had a favorable prognosis, even though the tumor was grade-3 endometrioid [173,174,177,181].

Certain molecular subtypes are associated with more rapid recurrence when treated with minimally invasive surgery. Both microsatellite-stable endometrioid tumors and p53-mutant tumors were associated with shorter recurrence-free survival when treated with minimally invasive surgery [135,182].

## 8. Concluding Remarks and Future Direction

**Figure 1:**
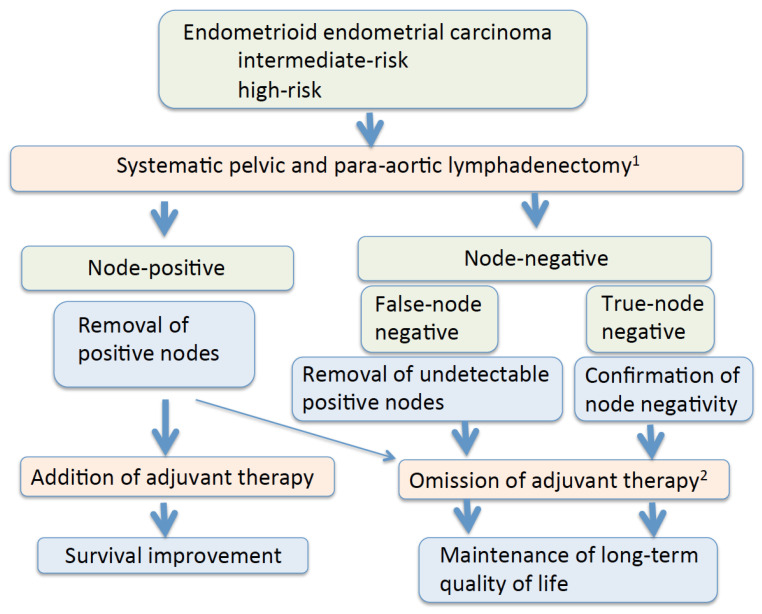
Possible therapeutic benefits of systematic lymphadenectomy. ^1^ Lymphadenectomy may cause surgical morbidity and lymphedema. ^2^ Omission of adjuvant therapy may be possible in patients with node-negative uterine-confined disease and patients with isolated tumor cells.

The most important goal of cancer therapy is cure. Maintenance of the long-term quality of life of survivors is another important goal. Lower extremity lymphedema was observed in 4.6–28.3% of endometrial cancer patients who underwent lymphadenectomy with or without adjuvant therapy [183,184,185,186] (Table 4). Of note, 3.4% of the patients who did not undergo lymphadenectomy developed lymphedema after endometrial cancer therapy [186]. As the use of postoperative radiation is associated with lower-extremity lymphedema, omission of radiation in node-negative patients decreases the incidence of lymphedema [187,188]. Removal of circumflex iliac nodes to the distal external iliac nodes is associated with the development of lymphedema, hence elimination of their dissection may be helpful [189]. In patients treated with surgery alone, lymph node staging procedures did not seem to affect quality of life or symptoms, suggesting that lymph node removal seems justified from the patient’s viewpoint [19]. Additionally, as external beam radiation may increase the risk of secondary cancer in the irradiated field, its use should be restricted, particularly in younger women [7,190]. In patients with intermediate- and high-risk endometrioid endometrial carcinomas, omitting unnecessary adjuvant therapy by performing systematic lymphadenectomy is a therapeutic benefit of lymphadenectomy (Figure 1).

Treatment guidelines for endometrial cancer based on the data from studies where a high percentage of patients had low-risk tumor cannot apply to patients with intermediate- and high-risk tumors. We believe open surgery with lymphadenectomy should be performed for patients with intermediate- and high-risk endometrioid endometrial carcinomas, and para-aortic lymphadenectomy is necessary for those with deep myometrial invasion [26]. To minimize the risk of lymph node recurrence, low-risk patients should also undergo lymph node assessment, excluding low-risk patients with tumors ≤ 2 cm who have a 0.6–0.8% risk of lymph node metastasis [191,192]. In addition, although postsurgical pathological examination may reveal lymphovascular space involvement that is an important risk factor of decreased survival [193,194], lymphovascular space involvement did not appear to reduce survival when lymph node metastasis was not detected [26,112,113,114,195]. These studies suggest that in endometrioid endometrial carcinoma, lymph node status determined by systematic lymphadenectomy separates patients into high-risk, i.e., node-positive, and low-risk, i.e., node-negative, groups [26,196].

The possibility of omitting adjuvant therapy in intermediate- and high-risk uterine-confined endometrioid endometrial carcinoma should be evaluated with real-world data and prospective randomized trials, comparing systematic lymphadenectomy with sentinel lymph node mapping, as well as comparing open surgery with minimally invasive surgery.

## Figures and Tables

**Table 1:** Low-, intermediate-, high-intermediate, and high-risk endometrioid endometrial carcinoma without extrauterine disease.

	Authors (Year)	Risk Group	Criteria
JSGO	Ebina (2016) [10]	Low	Grade 1 or 2, Stage IA
		Intermediate	Grade 1 or 2, Stage IB
			Grade 3, Stage IA
			Lymphovascular space invasion (LVSI)
		High	Grade 3, Stage IB
			Cervical stromal invasion
ESMO	Colombo (2013) [11]	Low	Grade 1 or 2, Stage IA
		Intermediate	Grade 3, Stage IA
			Grade 1 or 2, Stage IB
		High	Grade 3, Stage IB
PORTEC-1	Creutzberg (2000) [5]	Low	Grade 1, Stage IA
		Intermediate	Grade 1, Stage IB
			Grade 2, Stage IA and IB
			Grade 3, Stage IA
		High-intermediate	Age > 60 years + Grade 1 or 2 + Stage IB
			Age >60 years + Grade 3 + Stage IA
GOG-99	Keys (2004) [6]	Low	Grade 1 or 2, Stage IA
		Low-intermediate	Age ≤ 50 years + ≤ 2 pathological risk factors
			Age 50–69 years + ≤ 1 pathological risk factor
			Age ≥ 70 years + no pathological risk factor
			Risk factors (1) grade 2 or 3 histology,
			(2) positive LVSI
			(3) myometrial invasion to outer third
		High-intermediate	Any age + 3 pathological risk factors
			Age 50–69 years + ≥ 2 pathological risk factor
			Age ≥ 70 years + ≥ 1 pathological risk factor
ESGO/ ESTRO/ESP	Coucin (2021) [12]	Low	Grade 1 or 2, Stage IA, LVSI negative or focal
		Intermediate	Grade 1 or 2, Stage IB, LVSI negative or focal
			Grade 3, Stage IA, LVSI negative or focal
		High-intermediate	Stage I, substantial LVSI
			Grade 3, Stage IB, regardless of LVSI status
			Stage II

JSGO, Japan Society of Gynecologic Oncology; ESMO, European Society of Medical Oncology; GOG, Gynecologic Oncology Group; ESGO, European Society of Gynecological Oncology; ESTRO, European Society for Radiotherapy and Oncology; ESP, European Society of Pathology.

**Table 2:** Long-term outcomes of patients with intermediate- and high-risk endometrial carcinoma treated with open surgery alone, including pelvic and para-aortic lymphadenectomy.

Author (Year)	No. of Patients	Stage, Grade; Histology	Lymphadenectomy (LA)	No. of Nodes Removed	5-Year Survival Rate	Median Follow-Up
Chen (1989) [113]	18	IAG3, IB	Selective biopsy of pelvic and para-aortic lymph nodes	Not available	100% (DFS)	5–13 years
Ayhan (2002) [114]	25	IAG3, IB; endometrioid	Pelvic and para-aortic LA	27, median	92% (OS)	96 months
Straughn (2003) [115]	121	IB; serous and clear cell were excluded	Pelvic and para-aortic LA	20, mean	92% (OS)	41 months
Otsuka (2022) [26]	77	IAG3, IB, II; endometrioid	Pelvic LA in all patients and para-aortic LA in selected patients	19 (pelvic) 8 (para-aortic), median	97% (DSS)	75 months

DFS, disease-free survival; OS, overall survival; DSS, disease-specific survival.

**Table 3:** Effects of pelvic and para-aortic lymphadenectomy by age.

Author (Year)	No. of Patients	Lymphadenectomy	Number of Nodes Removed	Age	Tumor Type
*Positive effects of PLA/PALA on survival*					
Huang (2013) [99]	961	PLA, PALA	18 (pelvic) 5 (para-aortic)	53 y, median	Endometrioid
Todo (2010) [98]	671	PLA, PALA	59 (pelvic) 23 (para-aortic)	56 y, median	Other than low-risk (pT1A, G1-2)
Abu-Rustum (2008) [106]	1035	PLA, PALS (up to IMA)	16	61 y, median	Endometrioid
Mariani (2000) [95]	137	PLA, PALA	16 (pelvic) 6 (para-aortic)	67 y, mean	Patients at high risk for para-aortic lymph node involvement *
Eggemann (2016) [100]	1502	PLA, PALA	19	66 y (PLA and PALA) 68 y (PLA) 72 y (no LA), mean	Other than low-risk (pT1A, G1-2)
*No effects of PLA/PALA on survival*					
Papathemelis (2018) [129]	299	PLA, (PALA)	26	69 y, median	pT1B G1-2, type I
Ignatov (2020) [128]	2392	PLA, PALA	29	69 y (LA) 74 y (no LA), median	Endometrioid, intermediate-risk (pT1A G3, pT1B G1-2) high-risk (pT1B G3, pT2 Gany)

PLA, pelvic lymphadenectomy; PALA, para-aortic lymphadenectomy; PALS, para-aortic lymph node sampling; IMA, inferior mesenteric artery; NA, not available. * Myometrial invasion >50%, macroscopically positive pelvic nodes or positive adnexae, excluding stage IV disease.

**Table 4:** Incidence of lower extremity lymphedema and lymphocele in patients with endometrial cancer.

Author (Year)	No. of Patients	Lymphadenectomy (LA)	No. of Nodes Removed	Incidence of Lower Extremity Lymphedema	Incidence of Lymphocele
Menderes (2015) [185]	238	Robotically assisted PLA	16 (mean)	4.6 %	NA
Ghezzi (2012) [184]	138	Open PLA	18	14.6%	15.4%
		Laparoscopic PLA		13%	1.4%
Konno (2011) [183]	142	Open PLA	36 (median)	28.3%	9.4%
	138	Open PLA + PALA	87 (median)	23.2%	9.2%
Wedin (2020) [186]	116	PLA or PLA + PALA	25.4 (PLA), 38.4 (PLA + PALA)	15.8%	4.3%
	119	no LA		3.4%	0%

PLA, pelvic LA; PALA, para-aortic LA.
